# Cumulative incidence and risk factors for medication-related osteonecrosis of the jaw during long-term prostate cancer management

**DOI:** 10.1038/s41598-024-64440-7

**Published:** 2024-06-11

**Authors:** Masaru Tani, Koji Hatano, Akihiro Yoshimura, Yuki Horibe, Yutong Liu, Nesrine Sassi, Toshiki Oka, Yohei Okuda, Akinaru Yamamoto, Toshihiro Uemura, Gaku Yamamichi, Yu Ishizuya, Yoshiyuki Yamamoto, Taigo Kato, Atsunari Kawashima, Norio Nonomura

**Affiliations:** Department of Urology, Osaka University Graduate School of Medicine, 2-2 Yamadaoka, Suita, Osaka 565-0871 Japan

**Keywords:** Denosumab, Jaw, Osteonecrosis, Prostate cancer, Zoledronic acid, Medical research, Risk factors, Urology

## Abstract

Bone-modifying agents (BMA) are extensively used in treating patients with prostate cancer with bone metastases. However, this increases the risk of medication-related osteonecrosis of the jaw (MRONJ). The safety of long-term BMA administration in clinical practice remains unclear. We aimed to determine the cumulative incidence and risk factors of MRONJ. One hundred and seventy-nine patients with prostate cancer with bone metastases treated with BMA at our institution since 2008 were included in this study. Twenty-seven patients (15%) had MRONJ during the follow-up period (median, 19 months; interquartile range, 9–43 months). The 2-year, 5-year, and 10-year cumulative MRONJ incidence rates were 18%, 27%, and 61%, respectively. Multivariate analysis identified denosumab use as a risk factor for MRONJ, compared with zoledronic acid use (HR 4.64, 95% CI 1.93–11.1). Additionally, BMA use at longer than one-month intervals was associated with a lower risk of MRONJ (HR 0.08, 95% CI 0.01–0.64). Furthermore, six or more bone metastases (HR 3.65, 95% CI 1.13–11.7) and diabetes mellitus (HR 5.07, 95% CI 1.68–15.2) were risk factors for stage 2 or more severe MRONJ. MRONJ should be considered during long-term BMA administration in prostate cancer patients with bone metastases.

## Introduction

Almost half the patients with prostate cancer with bone metastases experience skeletal-related events (SREs) within 2 years, and preventing SREs is essential because they significantly reduce the quality of life^1^. Bone-modifying agents (BMAs), such as zoledronic acid and denosumab, are efficacious in reducing the risk of SREs in patients with castration-resistant prostate cancer (CRPC). Therefore, their use is recommended in patients with CRPC with bone metastases^2^. One of the most crucial adverse events of long-term BMA use is medication-related osteonecrosis of the jaw (MRONJ). It was first reported in 2003^3^ and is an intractable disease that negatively affects the quality of life.

More than a decade has passed since the routine use of BMAs began, and the Japanese Association of Oral and Maxillofacial Surgeons (JAOMS) has reported a significant rise in the incidence of MRONJ in clinical practice, a trend observed globally^4^. The JAOMS surveys have indicated a concurrent increase in the proportion of severe MRONJ cases^5,6^. Nashi et al. reported that the incidence of MRONJ is increasing due to prolonged treatment periods, and the number of patients with severe MRONJ of stage 2 or higher is also increasing^5^. However, the frequency of MRONJ varies considerably. Previous studies have documented an incidence of MRONJ ranging from approximately 0.1 to 32%^7–15^. Differences in the duration of observation and patient backgrounds may have influenced variations in the incidence of MRONJ. Survival time after BMA use appears to be associated with the incidence of MRONJ ^11^. However, long-term observational studies are limited.

MRONJ is a multifactorial disease and its etiology is not fully elucidated^10^. In addition to patient-related factors such as oral hygiene and diabetes, the type of BMA and dosing interval are important factors in the development of MRONJ^10,13^. A meta-analysis of 6 randomized controlled trials found a significantly higher incidence of MRONJ with denosumab compared to zoledronic acid over 3 years^7^. Additionally, a meta-analysis of 3 randomized trials indicated a lower incidence of adverse events, including MRONJ, when zoledronic acid was administered every 12 weeks rather than every 4 weeks^16^. However, the effects of BMA type and dosing interval on MRONJ remain unknown in cases treated with BMA for the long term.

The incidence of MRONJ in patients with prostate cancer (5–39%) has been reported to be higher than in patients with other cancers^1,11,17,18^. Patients with advanced prostate cancer now achieve prolonged survival owing to novel drugs and treatment modalities^19–21^. As a result, it is expected that the number of cases of long-term use of BMA would increase. However, the association between prostate cancer and a higher incidence of MRONJ remains unclear, and the risk factors for MRONJ have not been thoroughly discussed. Therefore, this study aimed to determine the cumulative incidence of MRONJ with the long-term use of BMAs in patients with prostate cancer with bone metastasis. We further made the null hypothesis that BMA type and dosing interval do not affect the occurrence of MRONJ. We also evaluate the cumulative incidence of stage 2 or more severe MRONJ and risk factors for MRONJ development.

## Materials and methods

### Study design and data collection

This was a retrospective, observational study conducted at a single center to determine the cumulative incidence and risk factors for MRONJ in prostate cancer patients with bone metastases who were treated with BMAs.

**Figure 1:**
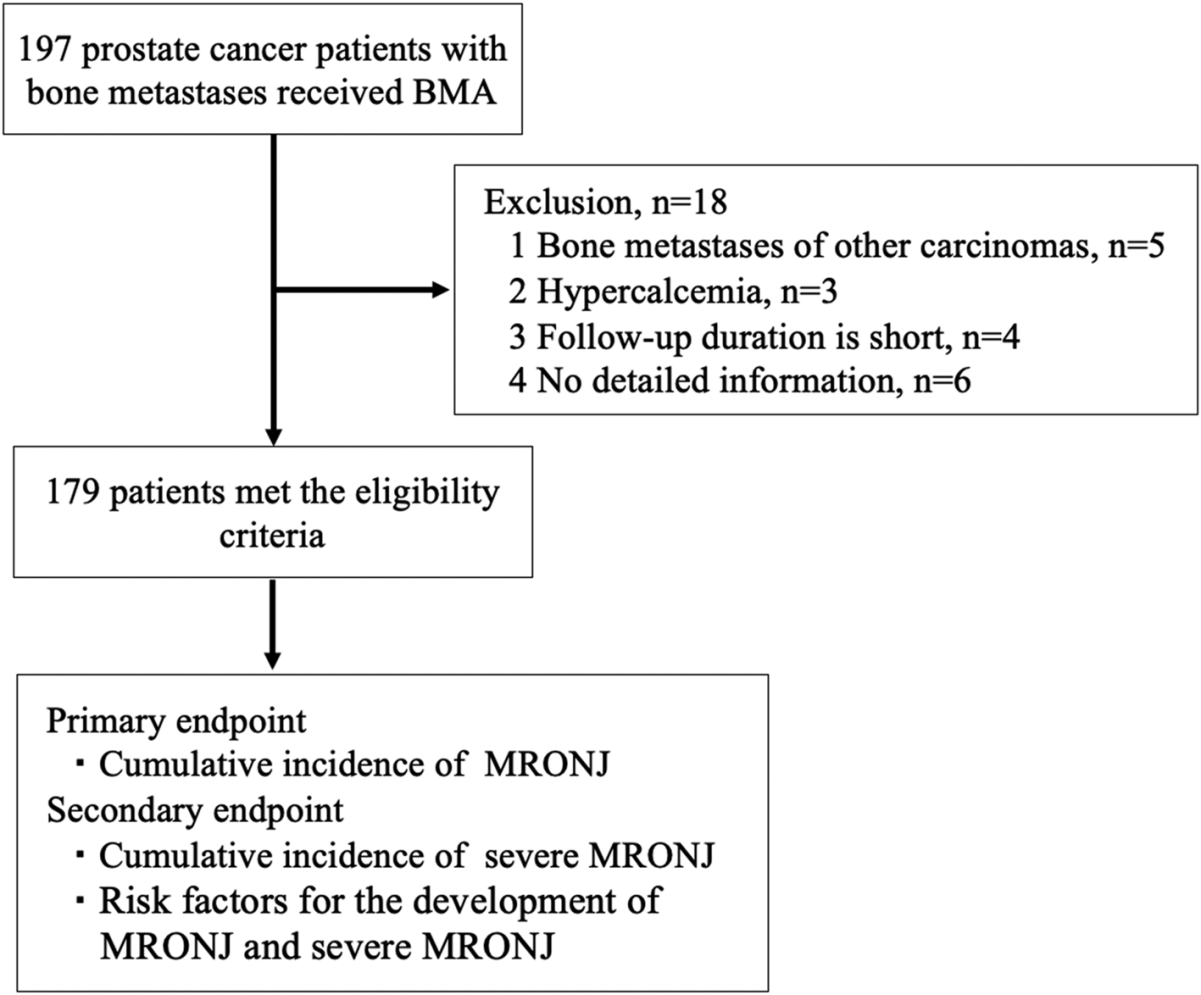
Study schematic.

The Department of Urology at our hospital has been using zoledronic acid since January 2008 and denosumab since July 2012 for patients with castration-sensitive or castration-resistant prostate cancer with bone metastases. In Japan, the utilization of zoledronic acid and denosumab for solid tumors with bone metastases is approved by the Japanese Drug Authority. Since July 2012, the attending physicians selected either zoledronic acid or denosumab, depending on the patient's preference and general condition. This study included 197 prostate cancer patients with bone metastases who were treated with BMAs between January 2008 and April 2023. Patients were enrolled at least 2 months after BMA administration. Patients who received BMAs for bone metastases from other carcinomas (n = 5) or for whom BMAs were used to treat hypercalcemia (n = 3) or for whom adequate clinical data were unavailable (n = 6) were excluded from the study. Ultimately, 179 patients met the study eligibility criteria (Fig. 1). Dental evaluation and care were discussed with the primary dentist before the initiation of BMAs. Zoledronic acid was administered intravenously at a dose of 4 mg every four weeks, and the dose was modified according to the manufacturer’s instructions for patients with renal dysfunction. Denosumab was administered subcutaneously at a dose of 120 mg every four weeks. Physicians were allowed to extend the intervals of zoledronic acid and denosumab administration to 6–12 weeks at the start of BMA therapy. In principle, zoledronic acid and denosumab were continued, with discontinuation criteria being the occurrence of MRONJ or deterioration of the patient's Performance Status.

### Endpoints

The primary and secondary endpoints were the cumulative incidence of MRONJ and the occurrence of severe MRONJ, respectively. The risk factors for the development of MRONJ and severe MRONJ were also examined. When comparing the BMAs for analysis, the first-line BMA received by the patient was selected.

MRONJ was diagnosed by dentists using the diagnostic criteria defined by the Japanese Allied Committee on Osteonecrosis of the jaw. The diagnosis was based on current or previous treatment with bisphosphonates or denosumab, the presence of exposed necrotic bone in the maxillofacial region lasting > 8 weeks, and no history of radiation therapy to the jawbone. The stage of MRONJ was indicated according to the 2017 Japanese Position Paper^22^, depending on the severity of symptoms. In this study, we defined severe MRONJ as MRONJ of stages 2 and 3.

We conducted analyses using data extracted from medical records to investigate the risk factors associated with MRONJ. The clinical parameters examined included: age, body mass index (BMI), type and duration of BMA treatment, BMA administration interval, prostate cancer type (castration-sensitive prostate cancer [CSPC] or CRPC), number of bone metastases at BMA initiation (extent of disease [EOD] grade^23^), presence of diabetes, medications (steroids and anticoagulants), and clinical laboratory data at BMA initiation (serum prostate-specific antigen [PSA], serum alkaline phosphatase [ALP], serum lactate dehydrogenase [LDH], serum calcium [Ca], serum albumin [Alb], hemoglobin [Hb], serum tartrate-resistant acid phosphatase-5b [TRACP-5b], serum bone-specific alkaline phosphatase [BAP], and serum pyridinoline cross-linked carboxyterminal telopeptide of type 1 collagen [ICTP] levels).

### Statistical analysis

The cumulative incidence of MRONJ was calculated using Kaplan–Meier analysis. Risk factors for MRONJ were analyzed by Cox regression analysis using JMP software (JMP version 17.0.0; SAS Institute Inc., Cary, NC, USA). Statistical significance was set at *p* < 0.05.

### Ethical approval

This study was approved by the Institutional Review Board of Osaka University (ethics review number: 13397-20). An opt-out consent procedure was employed for the use of patient data obtained in routine medical care. The informed consent was obtained from all participants prior to the initiation of BMA administration. The study was conducted in compliance with the Declaration of Helsinki.

### Consent to participate

Informed consent was obtained from all participants included in the study.

## Results

### Patient characteristics

The patient characteristics (n = 179) are summarized in Table 1. A total of 109 patients received zoledronic acid, and 70 patients received denosumab. Four patients who switched from zoledronic acid to denosumab during the study period were included in the zoledronic acid group: zoledronic acid duration (9, 17, 36, 36 months) and subsequent denosumab duration (37, 4, 4, 10 months). The median age of the patients was 72 years (interquartile range [IQR] 67–78), and the median observation period was 19 months (IQR 9–43 months). The treatment duration was longer, and the ALP, LDH, and BAP levels were higher at BMA induction in the zoledronic acid group than in the denosumab group. The Ca level at BMA induction was higher in the denosumab group than in the zoledronic acid group (Table 1).

**Table 1:** Patient’s characteristics at BMA induction.

	Total	Zoledronic acid	Denosumab	*p* value
	N = 179	N = 109 (61%)	N = 70 (39%)	
Median age, years (IQR)	72 (67–78)	71 (65–78)	73 (68–77)	0.606
Median BMI, kg/m^2^ (IQR)	22.0 (19.5–24.4)	22.0 (20.0–24.9)	22.1 (18.6–24.2)	0.179
Median duration of BMA treatment, months (IQR)	13 (7–28)	17 (9–47)	10 (5–22)	0.002
Median duration of ADT before BMA, months (IQR)	20 (2–54)	15 (1–50)	23 (3–56)	0.099
Median number BMA doses	11 (5–26)	15 (7–36)	9 (4–15)	< 0.001
Castration sensitivity, N (%)				
CSPC	72 (40%)	47 (43%)	25 (36%)	0.873
CRPC	107 (60%)	62 (57%)	45 (64%)	0.203
BMA agent, N (%)				
Zoledronic acid	109 (61%)			
Denosumab	70 (39%)			
EOD grade at BMA induction, N (%)				
0	4 (2%)	3 (3%)	1 (1%)	
1	64 (36%)	42 (39%)	22 (32%)	
2	55 (31%)	32 (29%)	23 (33%)	
3	38 (21%)	22 (20%)	16 (23%)	
4	16 (9%)	9 (8%)	7 (10%)	
Unknown	2 (1%)	1 (1%)	1 (1%)	
Dosing interval of BMA				
1 month	155 (87%)	97 (89%)	58 (83%)	0.244
> 1 month	24 (13%)	12 (11%)	12(17%)	0.239
Blood test results at BMA induction				
Median serum PSA, ng/ml (IQR)	25.9 (4.0–155)	38.2 (4.8–204.2)	15.2 (3.1–115.7)	0.21
Median serum ALP, U/l (IQR)	280 (182–502)	328 (222–581)	196 (112–400)	< 0.001
Median serum LDH, U/l (IQR)	200 (176–243)	207 (183–254)	190 (169–230)	0.003
Median serum Ca, mg/dl (IQR)	9.0 (8.7–9.4)	8.9 (8.5–9.2)	9.3 (8.9–9.6)	< 0.001
Median serum Alb, g/dl (IQR)	3.9 (3.5–4.1)	3.8 (3.4–4.2)	3.9 (3.6–4.1)	0.327
Median serum Hb, g/dl (IQR)	12.2 (11.0–13.2)	12.2 (11.0–13.3)	12.2 (10.9–3.1)	0.533
Median serum TRACP-5b, mU/dl (IQR)	541 (377–1027)	569 (401–1055)	395 (315–960)	0.204
Median serum BAP, μg/l (IQR)	19.8 (12.9–39)	24.1 (14.1–44.6)	14.6(11.8–21.9)	0.016
Median serum ICTP, ng/ml (IQR)	6.3 (4.2–11.7)	6.4 (4.2–11.4)	5.8 (4.0–12.2)	0.932
Corticosteroid usage, N (%)	82 (45%)	52 (47%)	30 (42%)	0.542
Diabetes mellitus, N (%)	34 (19%)	21 (19%)	13 (18%)	0.858
Anticoagulant usage, N (%)	42 (23%)	25 (23%)	17 (24%)	0.908

*BMA* bone-modifying agent; *BMI* body mass index; *ADT* androgen-deprivation therapy; *CSPC* castration-sensitive prostate cancer; *CRPC* castration-resistant prostate cancer; *EOD* extent of disease; *PSA* prostate-specific antigen; *ALP* alkaline phosphatase; *LDH* lactate dehydrogenase; *Ca* calcium; *Alb* albumin; *Hb* hemoglobin; *TRACP-5b* tartrate-resistant acid phosphatase-5b; *BAP* bone-specific alkaline phosphatase; *ICTP* pyridinoline cross-linked carboxy-terminal telopeptide of type 1 collagen.

### Cumulative incidence of MRONJ

**Figure 2:**
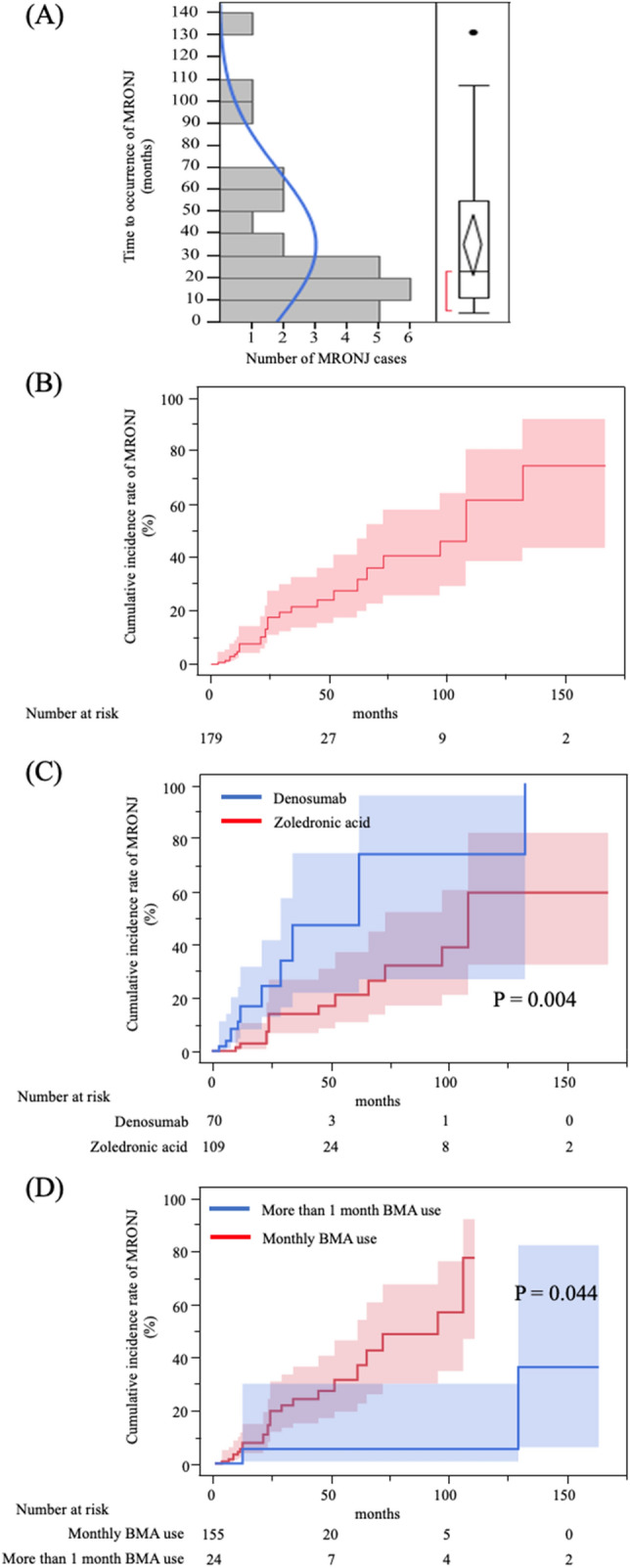
Cumulative incidence rate of any stage of MRONJ. (**A**) While the majority of cases of MRONJ occurred within three years, there were also several instances of MRONJ occurring during long-term treatment. (**B**) The 2-year, 5-year, and 10-year cumulative incidence rates of MRONJ were 18%, 27%, and 61%, respectively. (**C**) In the patients receiving zoledronic acid or denosumab, denosumab use rather than zoledronic acid use was a significant risk factor for the development of MRONJ (*p* = 0.004). (**D**) In the patients with monthly BMA use or BMA use at longer than one-month intervals, BMA administration at longer than one-month intervals was associated with a lower risk of MRONJ than monthly BMA use (*p* = 0.044). *MRONJ* medication-related osteonecrosis of the jaw.

MRONJ was diagnosed in 27 of 179 patients (15%) during the observation period, in 14 of 109 patients (13%) in the zoledronic acid group and 13 out of 70 patients (19%) in the denosumab group. The median duration of BMAs treatment before the onset of MRONJ was 23 months for all cohorts, with 25 and 13 months in the zoledronic acid and denosumab groups, respectively (Table 2). The MRONJ stages at diagnosis were 0 (15%), 1 (19%), 2 (44%), and 3 (22%) (Table 2). The time to occurrence of MRONJ was not normally distributed, with the majority of cases occurring within the first 3 years of treatment, although occurrences were also observed after long-term treatment (Fig. 2A). The cumulative incidence rates of MRONJ of any stage were 8%, 18%, 27%, and 61% at 1, 2, 5, and 10 years, respectively (Fig. 2B).

**Table 2:** Cumulative incidence of MRONJ.

	Total	Zoledronic acid	Denosumab
	N = 179	N = 109	N = 70
Characteristics of MRONJ			
Number of MRONJ cases (%)	27/179 (15%)	14/109 (13%)	13/70 (19%)
Median time to MRONJ, months (range)	23 (4–131)	25 (10–107)	13 (4–131)
MRONJ stage, N (%)			
0	4 (15%)	0 (0%)	4 (31%)
1	5 (19%)	3 (22%)	2 (15%)
2	12 (44%)	9 (64%)	3 (23%)
3	6 (22%)	2 (14%)	4 (31%)

*MRONJ* medication-related osteonecrosis of the jaw.

### Risk factors for the development of MRONJ of any stage

Table 3 presents the results of the analysis of factors associated with the development of MRONJ of any stage. In the univariate analysis, denosumab use rather than zoledronic acid use was a significant risk factor for the development of MRONJ (HR 3.26, 95% CI 1.46–7.24, *p* = 0.004). BMA administration at intervals longer than one month was associated with a lower risk of MRONJ than monthly BMA use (HR 0.13, 95% CI 0.02–0.95, *p* = 0.044). In the multivariate analysis, denosumab use was a significant predictor of MRONJ (HR 4.64, 95% CI 1.93–11.1, *p* < 0.001). In addition, BMA administration at longer than one-month intervals was associated with a lower risk of MRONJ (HR 0.08, 95% CI 0.01–0.64, *p* = 0.017). Kaplan–Meier curves are developed for the denosumab and zoledronic acid groups as well as for the monthly BMA and BMA administration at longer than one-month intervals groups (Figs. 2C,D).

**Table 3:** Risk factors for any stage of MRONJ (univariate and multivariate Cox proportional hazard analyses).

	Univariate analysis (N = 179)			Multivariate analysis (N = 179)		
	HR	95% CI	*p* value	RR	95% CI	*p* value
Age						
≧ 70 vs. < 70	1.3	0.59–2.84	0.507			
BMI						
≧ 25 vs. < 25	1.97	0.85–4.58	0.13			
BMA						
Denosumab vs. zoledronic acid	3.26	1.46–7.24	0.004	4.64	1.93–11.1	< 0.001
Castration sensitivity						
CRPC vs. CSPC	1.45	0.67–3.15	0.338			
T ≧ 3 vs. ≦ 2	1.61	0.66–3.89	0.272			
N ≧ 1 vs. 0	1.38	0.56–3.38	0.477			
M 1 vs. 0	1.98	0.74–5.23	0.141			
Gleason score 9 ≧ vs. ≦ 8	1.25	0.42–3.69	0.666			
Duration of ADT before BMA (months)						
≧ 20 vs. < 20	1.07	0.49–2.33	0.854			
Interval of BMA (months)						
> 1 vs. 1	0.13	0.02–0.95	0.044	0.08	0.01–0.64	0.017
EOD grade at BMA induction						
≧ 2 vs. < 2	2.18	0.91–5.20	0.078			
Use of steroid						
Yes vs. No	0.94	0.40–2.21	0.895			
Use of anticoagulant						
Yes vs. No	1.06	0.42–2.65	0.892			
Diabetes mellitus	1.87	0.77–4.52	0.165			
PSA at BMA induction (ng/ml)						
≧ 25.0 vs. < 25.0	0.93	0.41–2.09	0.869			
ALP at BMA induction (U/l)						
≧ 260 vs. < 260	1.08	0.50–2.33	0.839			
LDH at BMA induction (U/l)						
≧ 220 vs. < 220	0.88	0.36–2.11	0.784			
Ca at BMA induction (mg/dl)						
≧ 9.0 vs. < 9.0	2.17	0.93–5.07	0.073			
Alb at BMA induction (g/dl)						
≧ 4.0 vs. < 4.0	1.23	0.532.81	0.624			
Hb at BMA induction (g/dl)						
≧ 12.2 vs. < 12.2	1.23	0.56–2.73	0.595			
TRACP-5b at BMA induction (mU/dl)						
≧ 590 vs. < 590	5.01	0.58–43.0	0.142			
BAP at BMA induction (μg/l)						
≧ 20 vs. < 20	1.08	0.31–3.77	0.895			
ICTP at BMA induction (ng/ml)						
≧ 5.24 vs. < 5.24	0.36	0.07–1.83	0.22			

*MRONJ* medication-related osteonecrosis of the jaw; *BMI* body mass index; *BMA* bone-modifying agent; *CRPC* castration-resistant prostate cancer; *CSPC* castration-sensitive prostate cancer; *ADT* androgen-deprivation therapy; *EOD* extent of disease; *PSA* prostate-specific antigen; *ALP* alkaline phosphatase; *LDH* lactate dehydrogenase; *Ca* calcium; *Alb* albumin; *Hb* hemoglobin; *TRACP-5b* tartrate-resistant acid phosphatase-5b; *BAP* bone specific alkaline phosphatase; *ICTP* pyridinoline cross-linked carboxyterminal telopeptide of type 1 collagen.

### Cumulative incidence and risk factors for severe MRONJ

**Figure 3:**
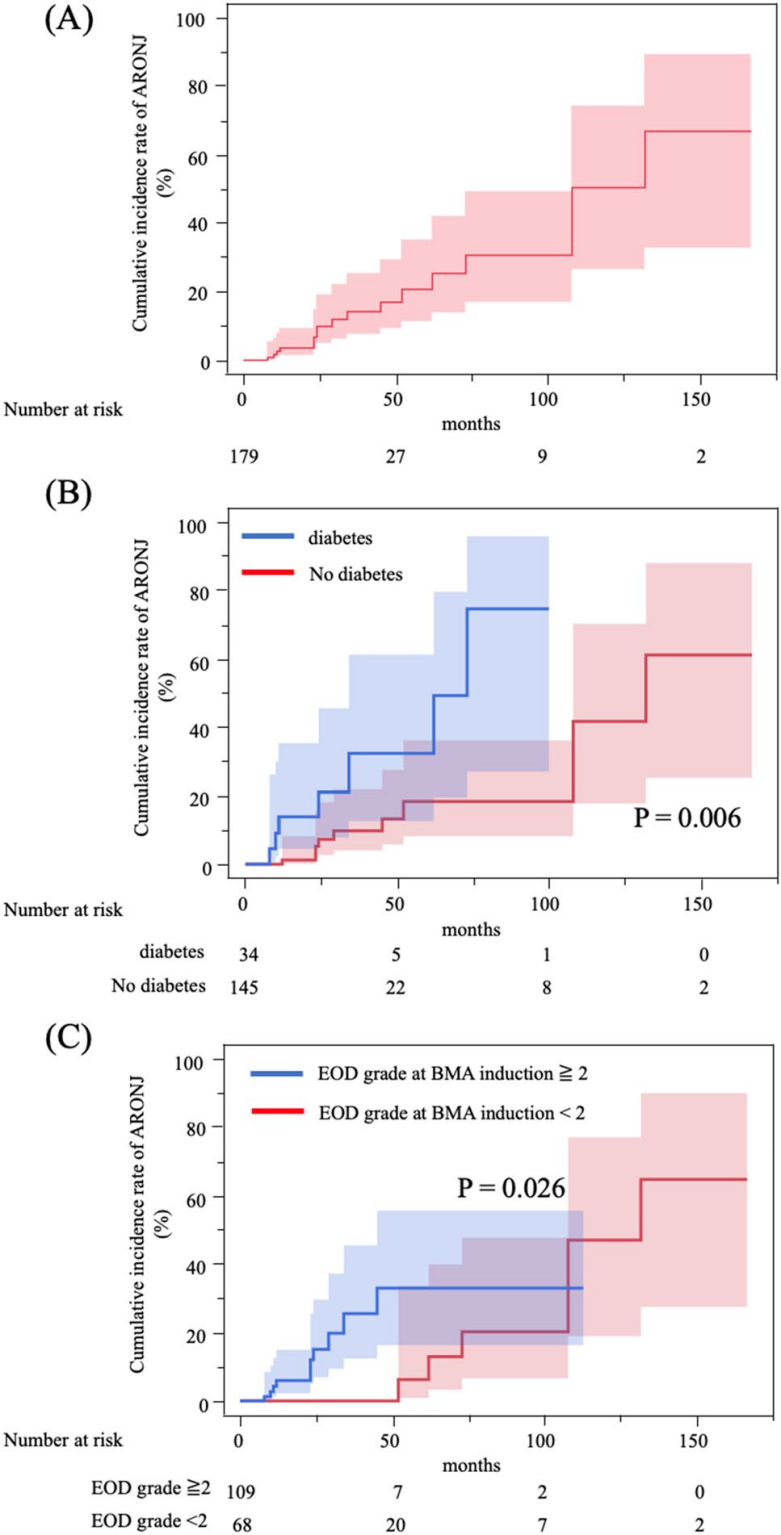
Cumulative incidence rate of severe MRONJ. (**A**) The 2-year, 5-year, and 10-year cumulative incidence rates of severe MRONJ were 7%, 21%, and 51%, respectively. (**B**) Among patients with or without diabetes, the incidence rate of severe MRONJ was significantly different between patients with diabetes and those without diabetes (*p* = 0.006). (**C**) Among patients with EOD grade at BMA induction ≧2 or EOD grade at BMA induction < 2, the incidence rate of MRONJ was significantly different between the patients with EOD grade ≧2 and those with EOD grade < 2 (*p* = 0.026). *MRONJ* medication-related osteonecrosis of the jaw; *EOD* extent of the disease.

The cumulative incidence rates of severe MRONJ were 3%, 7%, 21%, and 51% at 1, 2, 5, and 10 years, respectively (Fig. 3A). Additionally, Table 4 outlines the results of analyzing the factors associated with severe MRONJ of stage 2 or higher. In the univariate analysis, an EOD grade of 2 or higher at BMA induction (six or more bone metastases) compared to an EOD grade of 0 or 1 (HR 3.73, 95% CI 1.16–11.9, *p* = 0.026) and the presence of diabetes mellitus (HR 4.38, 95% CI 1.53–12.5, *p* = 0.006) were the significant risk factors for the occurrence of severe MRONJ. In the multivariate analysis, an EOD grade of 2 or higher at BMA induction (HR 3.65, 95% CI 1.13–11.7, *p* = 0.030) and the presence of diabetes mellitus (HR 5.07, 95% CI 1.68–15.2, *p* = 0.004) were the significant predictors of severe MRONJ (Fig. 3B,C).

**Table 4:** Risk factors for severe MRONJ (univariate and multivariate Cox proportional hazard analyses).

	Univariate analysis (N = 179)			Multivariate analysis (N = 179)		
	HR	95% CI	*p* value	RR	95% CI	*p* value
Age						
≧ 70 vs. < 70	1.1	0.41–2.90	0.846			
BMI						
≧ 25 vs. < 25	1.52	0.48–4.77	0.47			
BMA						
Denosumab vs. zoledronic acid	2.06	0.73–5.84	0.172			
Castration sensitivity						
CRPC vs. CSPC	1.43	0.54–3.76	0.468			
T ≧ 3 vs. ≦ 2	2.09	0.67–6.53	0.202			
N ≧ 1 vs. 0	1.06	0.32–3.48	0.92			
M 1 vs. 0	2.13	0.61–7.43	0.235			
Gleason score 9≧ vs. ≦8	1.07	0.30–3.80	0.915			
Duration of ADT before BMA (months)						
≧ 20 vs. < 20	0.73	0.26–2.04	0.56			
Dosing interval of BMA (months)						
> 1 vs. 1	2.23 × 10–9	0–NA	0.999			
EOD grade at BMA induction						
≧ 2 vs. < 2	3.73	1.16–11.9	0.026	3.65	1.13–11.7	0.03
Use of steroid						
Yes vs. No	0.529	0.14–1.88	0.895			
Use of anticoagulant						
Yes vs. No	1.58	0.55–4.54	0.391			
Diabetes mellitus	4.38	1.53–12.5	0.006	5.07	1.68–15.2	0.004
PSA at BMA induction (ng/ml)						
≧ 25.0 vs. < 25.0	1.27	0.46–3.51	0.641			
ALP at BMA induction (U/l)						
≧ 260 vs. < 260	0.76	0.28–2.00	0.58			
LDH at BMA induction (U/l)						
≧ 220 vs. < 220	0.78	0.24–2.44	0.67			
Ca at BMA induction (mg/dl)						
≧ 9.0 vs. < 9.0	1.33	0.48–3.66	0.578			
Alb at BMA induction (g/dl)						
≧ 4.0 vs. < 4.0	1.36	0.48–3.80	0.554			
Hb at BMA induction (g/dl)						
≧ 12.2 vs. < 12.2	1.04	0.39–2.77	0.935			
TRACP-5b at BMA induction (mU/dl)						
≧ 590 vs. < 590	4	0.44–36.0	0.215			
BAP at BMA induction (µg/l)						
≧ 20 vs. < 20	1.47	0.32–6.59	0.614			
ICTP at BMA induction (ng/ml)						
≧ 5.24 vs. < 5.24	0.747	0.12–4.62	0.755			

*MRONJ* medication-related osteonecrosis of the jaw; *BMI* body mass index; *BMA* bone-modifying agents; *CRPC* castration-resistant prostate cancer; *CSPC* castration-sensitive prostate cancer; *ADT* androgen-deprivation therapy; *EOD* extent of disease; *PSA* prostate-specific antigen; *ALP* alkaline phosphatase; *LDH* lactate dehydrogenase; *Ca* calcium; *Alb* albumin; *Hb* hemoglobin; *TRACP-5b* tartrate-resistant acid phosphatase-5b; *BAP* bone specific alkaline phosphatase; *ICTP* pyridinoline cross-linked carboxyterminal telopeptide of type 1 collagen.

## Discussion

This study focused on the cumulative incidence of MRONJ with long-term BMA use in prostate cancer patients with bone metastases. The cumulative incidence of MRONJ was approximately one-quarter at 5 years, but at 10 years, more than half of the patients had developed the disease. Denosumab use was the risk factor for MRONJ of any stage, whereas BMA administration at longer than one-month intervals was associated with a lower risk of MRONJ. Thus, the null hypothesis that BMA type and dosing interval do not affect the occurrence of MRONJ has been rejected. The study also found that the presence of diabetes and an EOD grade of 2 or higher at BMA induction were risk factors for severe MRONJ.

Recently, Nakai et al.^1^ reported an MRONJ incidence rate of 27.5% in patients with prostate cancer and bone metastases at 5 years, which is similar to our findings. However, the incidence at 10 years remained unknown. In this study, the 10-year cumulative incidence of MRONJ was 61%. Although the majority of MRONJ cases occurred within the first 3 years, some MRONJ cases were observed even after long-term treatment. This highlights the substantial risk associated with long-term BMA use. The association between prolonged BMA use and an increased incidence of MRONJ has been increasingly reported^10,11,24,25^, making the optimal duration of BMA administration a crucial issue for the future, especially in patients with prostate cancer who undergo prolonged BMA therapy.

Currently, 3–4-weekly zoledronic acid administration or 4-weekly denosumab administration is recommended to reduce the incidence of SREs in patients with CRPC with bone metastases. However, the optimal dosing intervals remain unclear. A systematic review of three randomized trials revealed a decreased occurrence of MRONJ, with zoledronic acid administered every 12 weeks compared to every 4 weeks^16^. In breast cancer, dosing intervals of zoledronic acid every 3–4 weeks and every 12 weeks are both recommended as first choices^26^. In prostate cancer, zoledronic acid and denosumab are recommended every 4 weeks according to the ASCO-CCO and ESMO guidelines^27,28^. However, given the increased incidence of MRONJ with prolonged administration of BMA, extending the dosing interval is an issue that should be considered. To date, there are no established views on extended BMA dosing intervals in prostate cancer. At our hospital, the dosing interval is currently extended based on the judgment of the clinician. This study found that patients with BMA administration intervals longer than one month had a lower risk of any stage of MRONJ than those that were administered BMAs every 4 weeks. Among patients who received BMA at intervals of longer than one month at the start of therapy, severe cases of MRONJ were rare (only one case). In patients who have been using BMAs for a long time, a decrease in the cumulative dose owing to longer dosing intervals may reduce the risk of developing MRONJ.

Although denosumab is more effective than zoledronic acid in reducing the incidence of SREs, an analysis of eight RCTs found a significantly higher incidence of MRONJ with denosumab than with zoledronic acid administration^7^. The incidence of MRONJ with denosumab use ranged from 0.5–2.1% at 1 year, 1.1–3.0% at 2 years, and 1.3–3.2% at 3 years^7^. Long-term denosumab use has been associated with an increased risk of MRONJ; however, observational studies on denosumab treatment beyond 3 years are limited. Our study identified denosumab use as an independent risk factor for MRONJ development compared with zoledronic acid use, even during long-term follow-up. The use of denosumab is expected to increase in the future because of its potential to mitigate nephrotoxicity and its non-requirement for intravenous infusion. The different degrees of osteoclast activity inhibition by zoledronic acid and denosumab may account for the differences in the incidence of MRONJ. In this study, there was a higher proportion of MRONJ stage 0 cases in the denosumab group. This could suggest that varying degrees of osteoclast inhibition led to an increase in non-exposed lesions, which do not exhibit bone exposure but show imaging evidence of bone resorption or osteonecrosis. However, the mechanisms underlying the increased risk of MRONJ associated with denosumab use remain unclear. When considering long-term BMA administration, it may be better to select zoledronic acid or increase the dosing interval for denosumab. A clinical trial investigating the incidence of MRONJ with 4-weekly denosumab administration versus 12-weekly administration is ongoing (NCT02051218), and its findings are eagerly anticipated.

This study revealed a 7% incidence of severe MRONJ at the 2-year mark, which escalated to a significant 51% cumulative incidence over 10 years. The American Association of Oral and Maxillofacial Surgeons (AAOMS) recently announced that the cumulative BMA dose was unrelated to severity, which differs from previous observations^29^. Reports of a higher incidence of severe MRONJ in patients receiving long-term zoledronic acid administration are consistent with the findings of the present study^5^. Depending on the disease stage, conservative or surgical therapy is recommended for MRONJ^29,30^. Many studies have reported better efficacy and outcomes in patients undergoing surgical therapy than in those who do not^31,32^, leading to a trend toward recommending surgical therapy. Palla et al. stated that early surgery has a higher cure rate, is less invasive, and is associated with a better quality of life than advanced-stage surgery^33^. Therefore, diagnosing MRONJ at an early stage is crucial.

Although the pathogenesis of MRONJ is not fully understood, it is believed to be related to the presence of numerous bacteria in the oral cavity and specific characteristics of the jawbone. Inflammation easily spreads to the jawbone because of tooth infection, and the jawbone is situated in an infection-prone area^34^. Furthermore, BMA administration, with its resultant bone remodeling inhibition, suppression of osteoclast activity, and angiogenesis inhibition combined with the abundance of oral bacteria and easily infected jawbones, is hypothesized to cause MRONJ^22,35^. Nashi et al. reported that age and low serum albumin levels were associated with severe MRONJ, reflecting delayed wound healing^36^. The carcinomatous state with a high number of bone metastases is a systemic inflammatory state in which inflammation delays wound healing and increases scar formation^37^. In this study, we found that the presence of numerous bone metastases with an EOD of grade 2 or higher at BMA induction was a risk factor for severe MRONJ. These results support the finding that MRONJ results from local inflammation that is associated with jawbone infection^38^ and becomes more severe due to delayed wound healing. In this study, diabetes was identified as a risk factor for severe MRONJ. Diabetes has been reported to be associated with delayed wound healing^37^, and obesity has been associated with an increased number of oral microorganisms, contributing to periodontal disease^37,38^ and poor oral hygiene^39,40^. These reports are consistent with our observation that diabetes is a risk factor for severe MRONJ.

This study had several limitations. Firstly, this was a single-center, retrospective, observational study with a relatively small number of patients who developed MRONJ, resulting in limited findings. Secondly, data on the outcomes of patients who developed MRONJ were lacking, making it unclear whether the disease improved, remained stable, or worsened. Thirdly, the MRONJ diagnoses were not made by a single dentist, introducing potential variability in the diagnostic process. Fourth, the assessment of MRONJ relied solely on AAOMS staging. Clinical symptoms such as pain and signs of infection, along with radiographical evaluation, were utilized to evaluate the disease, but some variability may exist.

## Conclusion

In conclusion, this study revealed a significantly increased cumulative incidence of MRONJ in patients with prostate cancer and bone metastases after long-term BMA treatment.

Denosumab use was identified as an independent risk factor for MRONJ, and BMA use at intervals longer than one month was associated with a lower risk of MRONJ. Diabetes mellitus and numerous bone metastases were also identified as risk factors for severe MRONJ. These findings advocate for considering extended BMA dosing intervals in patients with prostate cancer with bone metastases undergoing long-term therapy. Furthermore, the recognition of risk factors for severe MRONJ underscores the importance of early detection in patients with diabetes mellitus and multiple bone metastases as a preventive measure.

## Data Availability

Raw data were generated at Osaka University. Derived data supporting the results of this study are available upon request from the corresponding author.
